# Radiological evaluation in patients with clinical suspicion of cerebral venous sinus thrombosis presenting with nontraumatic headache - a retrospective observational study with a validation cohort

**DOI:** 10.1186/s12880-020-00426-x

**Published:** 2020-02-27

**Authors:** Håkan Almqvist, Michael Mazya, Alberto Falk Delgado, Anna Falk Delgado

**Affiliations:** 1 Department of Clinical Neuroscience, Karolinska Institutet, 17176 Stockholm, Solna Sweden; 2 Department of Neuroradiology, Karolinska University Hospital, 17176 Stockholm, Solna Sweden; 3 Department of Neurology, Karolinska University Hospital, Stockholm, Sweden; 4 Department of Surgical Sciences, Uppsala University, Uppsala, Sweden

**Keywords:** Cerebral venous sinus thrombosis (CVST), Diagnostic accuracy, Headache, High-attenuating, Nonenhanced computed tomography

## Abstract

**Background:**

Clinical suspicion of cerebral venous sinus thrombosis (CVST) is imprecise due to non-specific symptoms such as headache. The aim was to retrospectively assess the diagnostic value of nonenhanced CT (neCT) in patients with nontraumatic headache and clinically suspected CVST.

**Methods:**

A retrospective consecutive series of patients referred 2013–2015 for radiology were evaluated. Eligible patients had nontraumatic headache and suspicion of CVST stated in the referral, investigated with CT venography (CTV) and nonenhanced CT (neCT). neCT scans were re-evaluated for the presence of CVST or other pathology. All CTVs were checked for the presence of CVST. The validation cohort consisted of 10 patients with nontraumatic CVT (2017–2019).

**Results:**

Less than 1% (1/104) had a suspected thrombus on neCT, confirmed by subsequent CTV. The remaining 99% had a CTV excluding CVST. Eleven percent had other imaging findings explaining their symptoms. In the patient with CVST, the thrombosed dural sinus was high attenuating (maximum HU 89) leading to the suspicion of CVST confirmed by CTV. The validation cohort (*n* = 10) confirmed the presence of a high attenuating (HU > 65) venous structure in the presence of a confirmed thrombus in all patients presenting within 10 days (suspicion written in referral, 10%).

**Conclusions:**

Despite clinical suspicion, imaging findings of CVST in nontraumatic headache are uncommon. Evaluating neCT for high attenuation in dural sinuses, followed by CTV for confirmation in selected cases seems reasonable. CVST should be recognized by all radiologists and requires a high level of awareness when reading neCT for other indications.

## Background

Cerebral venous sinus thrombosis (CVST) is an uncommon disease mainly occurring in patients with specific risk factors, such as inborn or acquired coagulopathies and trauma [1–3]. CVST affects fewer than two individuals per 100,000 persons/year [4, 5] with headache as the most common symptom, present in up to 89% of patients [2, 6–10]. The clinical diagnosis is difficult, with radiological investigation required to confirm the diagnosis [10–16]. Correct clinical suspicion of CVST is complicated by the fact that the condition frequently causes symptoms also seen in more common diagnoses, such as stroke or brain tumors. Further, more benign conditions such as idiopathic intracranial hypertension can mimic symptoms of CVST.

Isolated headache is a common but unspecific symptom for patients seen in emergency departments and only a fraction of these patients have CVST. Specifically, patients presenting with acute headache at the emergency department are often young women [17]. Patient age is one important consideration when deciding imaging strategy, since computed tomography (CT) exposes the patient to ionizing radiation [18]. Previous studies have reported on the diagnostic utility of nonenhanced head computed tomography (neCT) to suspect CVST due to high density in the sinuses [11, 12, 19–22]. Despite previous reports suggesting the applicability of neCT to accurately identify acute CVST, other methods such as magnetic resonance imaging (MRI) or CT venography (CTV) are recommended to verify the diagnosis of CVST. Further, the use of a contrast-enhanced CTV only, would give incomplete evaluation of the cerebral parenchyma. MRI is a sensitive method for the evaluation of both brain parenchyma and cerebral vessels, but its limited availability for this indication restricts its potential to be a first order examination in many centers. Furthermore, MRI has a higher propensity for false positive diagnosis, with lower specificity [23]. Although considered the gold standard, digital subtraction angiography is not a common diagnostic tool for CVST.

At our department, patients with acute nontraumatic headache and a written referral to radiology asking to rule out or confirm CVST are primarily investigated with neCT with or without subsequent CTV, depending on the findings on neCT and the level of clinical suspicion for CVST. The level of clinical suspicion is based on clinical findings such as increased intracranial pressure, papilla oedema and known risk factors for CVST. The primary aim of this retrospective study was to assess imaging findings in patients with nontraumatic headache, a clinical suspicion of CVST primarily investigated with a neCT and subsequent CTV scans to establish the role for neCT as a screening tool for CVST. Study results were validated against imaging findings in a cohort of patients with confirmed CVT.

## Methods

This study was approved by the regional ethical review board and informed consent by study participants was waived due to the retrospective nature of the study. Consecutive CT scans of patients referred to a high volume neuroradiological department were extracted from the Picture Archiving and Communication System (PACS), and Radiology Information System (RIS) from January 2013 to December 2015. Patients were presenting to the emergency department staffed by specialists and residents in neurology. The retrospective patient cohort was validated against a cohort of ten patients with confirmed cerebral venous thrombosis (CVT) investigated between 2017 and 2019.

### Main cohort

#### Eligibility criteria

- Patient with a written imaging referral asking to rule out or confirm CVST in non-traumatic headache
- Radiological evaluation with neCT and CTV of the brain 2013–2015
- First time radiological examination for suspicion of CVST, onsite

#### Exclusion criteria

- Recent history of trauma or cervical, cranial or facial surgery (within 3 months)
- History of previous CVST

### Validation cohort

#### Eligibility criteria

- CVT or CVST
- neCT and CTV of the brain 2017–2019 performed for any indication
- Radiological examination onsite or offsite (transferred images *e g* due to patient transfer to our centre, or for radiological secondary review)
- Headache or other symptoms

#### Exclusion criteria

- Recent history of trauma or cervical, cranial or facial surgery (within 3 months)

### CT scan and blinded re-evaluation

neCT examinations were performed with a General Electric CT 750 High definition, 64-slice scanner with 120 kV, CT dose index volume (16 cm), approximately 45 mGy-cm, soft algorithm and 30% adaptive statistical iterative reconstruction (General Electric Healthcare, US, Chicago). Helical scanning mode, collimation 20 mm, pitch 0:5, rotation time 0.5 s, 0.625 mm slices without overlap, soft algorithm together with 3 plane reconstructions 5 mm without overlap saved routinely in the PACS. CTV was performed by injecting 100 cc of iodine contrast (iodexanol 320 mg I/mL) at 4 cc/sec followed by 80 cc of saline chaser. Craniocaudal scanning from vertex to the disk C2/C3. Submillimetre slice with 50% overlap (0.625/0.315). Helical scan 0.4 s rotation, 80 kVp, table movement 0:984, automA function with 175–420 mA at noise index 22. CTDIvol_16_ between 8 and 20 mGy. Dose-length-product (DLP) between 270 and 500 mGycm and DLP for the smartprep 10–35 mGycm. Manual start with minimum delay when the veins are clearly visible below the skull base with so called smart-prep technique.

Blinded for previously written report and patient symptoms, nonenhanced brain CT was re-evaluated in the local PACS by a specialist in Neuroradiology with 9 years of experience in brain CT evaluation. Scans were visually re-assessed for high density in cerebral dural sinuses on axial 0.63 mm thin slices and on reformatted 5 mm thick sagittal, axial, and coronal slices. Scans were evaluated for the presence of a suspected CVST or other imaging findings, including the presence of thrombus in the deep venous system. Visual inspection assessed heterogeneity in grey-scale within the intracranial venous structures and compared the grey-scale as appreciated in the dural sinuses compared to arterial structures and normal brain parenchyma. If a scan was reported positive for suspected CVST, a measurement of maximum Hounsfield units (HUs) in the suspected clot was also performed. Indeterminate test results were discussed with a second investigator with 16 years of experience in neuroradiology.

### Region of interest delineation

**Fig. 1:**
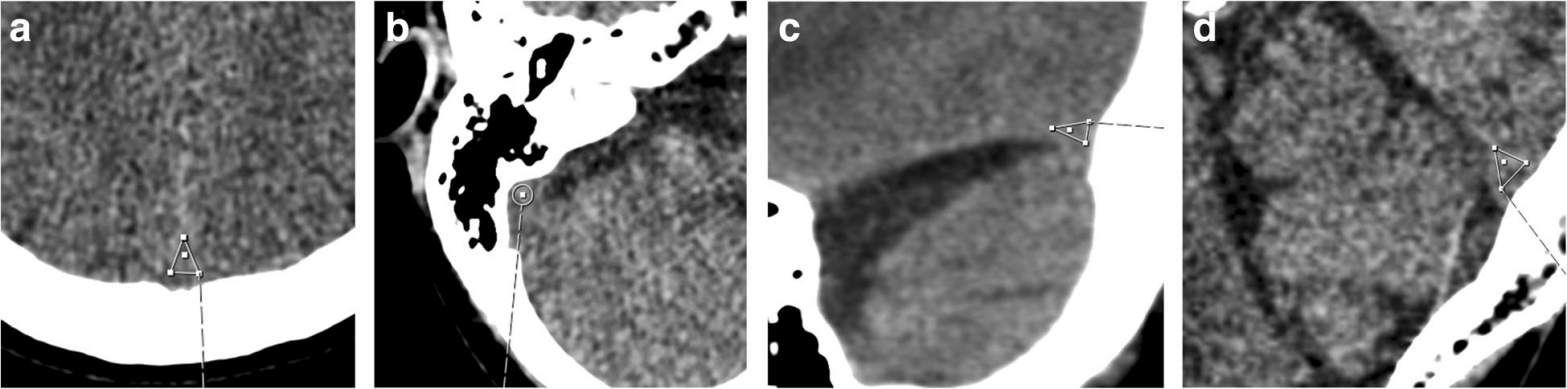
**a** Region of interest delineation in the superior sagittal sinus was performed on 0.63 mm thin axial slices at the level above the lateral ventricles as a triangular shape in the sinus. **b** Region of interest delineation in the left and right sigmoid sinus was performed on axial 0.63 mm thick slices at the level of the internal acoustic meatus in the sinus. Red arrow indicates region of measurement. **c** A region of interest (ROI) in the left and right transverse sinus was performed on reformatted 5 mm sagittal slices at the level just lateral to the lateral ventricles as a triangular shaped ROI. Red arrow indicates region of measurement. **d** A region of interest (ROI) in the torcular herophili was delineated on sagittal reformatted 0.63 mm thick slices

Standardized measurements of HU in cerebral dural sinuses were performed in the torcular herophili, superior sagittal sinus, transversal sinus, and sigmoid sinus bilaterally as shown in Fig. 1a–d in all included patients. In region of interest delineation, care was taken to avoid intravenous Paccioni granulations, dural structures, and beam hardening artifacts from nearby skull bone. Exact ROI position is detailed in the figure legends.

### Patient data extraction

After visual and quantitative evaluation of scans, the original radiology report was controlled for congruency with the blinded re-evaluation. In clinical routine, each original report had been double-signed by at least one specialist in general radiology and one experienced neuroradiologist. CTV findings and final diagnosis from patient charts were tabulated. Data on patient sex, age, and clinical information on risk factors for CVST were extracted from patient records. Laboratory reports were checked for hemoglobin and d-dimer values at the time of the radiological examination.

### Statistical analysis

Descriptive statistical methods were applied to summarize the extracted data. Data points were visually presented as scatter and box plots. Correlation analysis was performed with the Pearson’s correlation test. Statistical differences between groups were evaluated with Pearson’s Chi-2, Fisher’s exact test and Student T-test as appropriate. Receiver operating analysis was performed to assess diagnostic performance, optimal cut-off and sensitivity and specificity. Statistical analyses were performed in Statistica 12 (Dell Inc., Tulsa, OK, USA).

## Results

### Patient characteristics

In total, 104 patients (84 females, 81%) were included in the main study cohort. Mean (SD) patient age was 37 (12) years. Median (IQR) duration of headache was 7 (2–21) days. All patients in the main study cohort had been referred for a neuroradiological examination, been examined with both a neCT and CTV, had a medical history of nontraumatic headache, and a clinical suspicion from the referring physician of CVST written in the radiological referral. Referrals for head CT examination were from the emergency department in 81 cases (78%), inpatient wards in 18 cases (17%) and the outpatient neurology clinic in 5 cases (5%).

Ten patients were included in the validation cohort. Sixty percent (6/10) were evaluated at the same hospital as the main cohort while 40% (4/10) had their primary investigation in an outside hospital, with images digitally transferred for a second radiological review. Mean (SD) age was 47 (28) years (*p* ≥ 0.05 versus the main cohort). Six patients (60%) were female.

The proportion of females did not differ significantly between the main and validation cohort, *p* = 0.13.

### Symptom duration and risk factors main cohort

CVST (*n* = 1) was related to a symptom duration of headache for 3 days compared to unaffected individuals (*n* = 103), who had a median (IQR) symptom duration of headache of 7 (2–21) days. Out of 104 patients, 47% (49/104) had no known risk factor for venous thromboembolism (VTE) in the medical records or radiological referral. Fifty-three percent (55/104) had one or more reported risk factor of: current pregnancy (*n* = 2), recent pregnancy (*n* = 15), treatment with oral contraceptives (*n* = 3), ongoing in-vitro fertilization treatment (*n* = 1), history of VTE (*n* = 12), recent or current facial/ear/meningeal infection (*n* = 10), current oncological disease (*n* = 2), high hematocrit or polycythemia vera (*n* = 2), antiphospholipid syndrome (*n* = 4), Von Willebrand’s disease (*n* = 1), thalassemia minor (*n* = 1), heredity for pulmonary embolism (*n* = 1), and systemic inflammatory disease (*n* = 3). All included patients had imaging performed to rule out or confirm clinically suspected CVST.

### Symptom duration and risk factors, validation cohort

Median (IQR) symptom duration was 3 (1–7) days. Sixty percent (6/10) presented with headache. Other symptoms were loss of consciousness (*n* = 2), seizures (*n* = 1) and aphasia (*n* = 1). Only one patient (1/10 (10%)) had a clinical suspicion of CVST written in the referral (Table 2). The queries on the referrals included: hemorrhage? (*n* = 6), infarct? (*n* = 4), tumor? (*n* = 3), CVST? (n = 1), and dissection? (*n* = 1). Seventy percent had a known risk factor for thromboembolic events, compared to 58% in the main cohort (*p* = 0.34).

### NeCT and HU in unaffected and thrombosed sinuses, main cohort

**Fig. 2:**
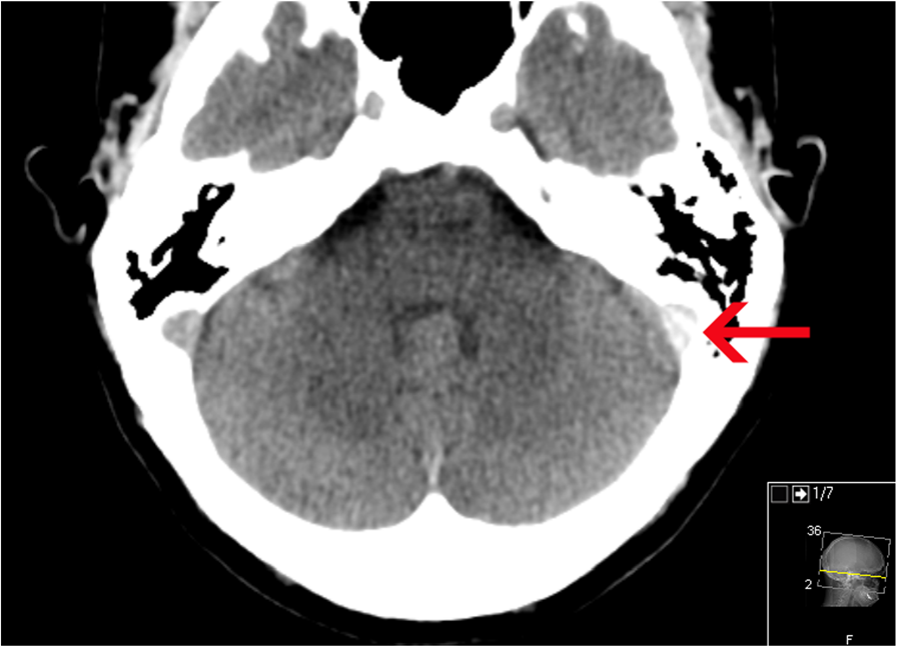
Axial reformatted 5 mm nonenhanced CT of suspected thrombus in the left sigmoid sinus marked by a red arrow (window width 90 window level 40). Note the normal HU value in the corresponding right sigmoid sinus

In blinded reevaluation, one patient (1/104, 1.0%) had suspicion of CVST presenting as an intradural high density (Fig. 2) with the highest HU in the left sigmoid sinus (HU = 89), while 103 patients (99.0%) had no suspicion of CVST on neCT.

**Fig. 3:**
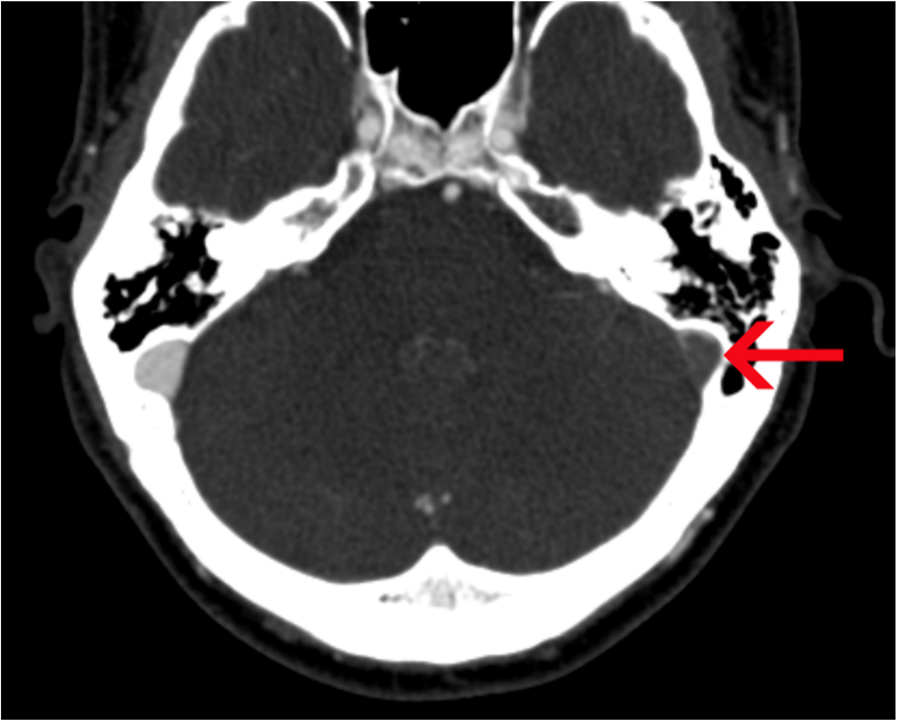
Axial multiplanar reconstruction (2 mm) of CTV verifying the thrombosed left sigmoid sinus indicated by a red arrow

Data on HU in unaffected sinuses in the 103 CVST-negative patients is presented in Table 1. Median HU in the torcular herophili was 52.0 (IQR 48.0–55.0), superior sagittal sinus 57 (IQR 54–60), 59 in left (IQR 55–64) and right (IQR 54–63) sigmoid sinuses and 57 (IQR 54–60) and 56 (IQR 54–59) in left and right transverse sinus. The maximum HU in the patient with suspected thrombus on neCT was 89 in the thrombus measured in the left sigmoid sinus (CVST confirmed by CTV as depicted in Fig. 3). An inverse correlation between HU and patient age was found, with HU decreasing with increasing age (*r* = − 0.20, *p* = 0.04). The variance of HU was highest in the sigmoid sinus (6.1–6.6 HU) and lowest in transverse sinuses and torcular herophili (4.1–4.5 HU).

**Table 1:** Hounsfield unit values from nonenhanced CT in dural sinuses in patients (*n* = 103) not diagnosed with cerebral sinus venous thrombosis

Descriptive Statistics	Mean (SD)	Median (IQR)	Minimum	Maximum
Hounsfield units in torcular herophili	51.7 (4.4)	52 .0 (48.0–55.0)	40.0	60.0
Hounsfield units in superior sagittal sinus	56.6 (4.7)	57.0 (54.0–60.0)	39.0	68.0
Hounsfield units in left sigmoid sinus	58.5 (6.6)	59.0 (55.0–64.0)	40.0	69.0
Hounsfield units in right sigmoid sinus	58.3 (6.1)	59.0 (54.0–63.0)	40.0	70.0
Hounsfield units in left transverse sinus	56.9 (4.1)	57.0 (54.0–60.0)	46.0	66.0
Hounsfield units in right transverse sinus	56.0 (4.5)	56.0 (54.0–59.0)	40.0	64.0

**Fig. 4:**
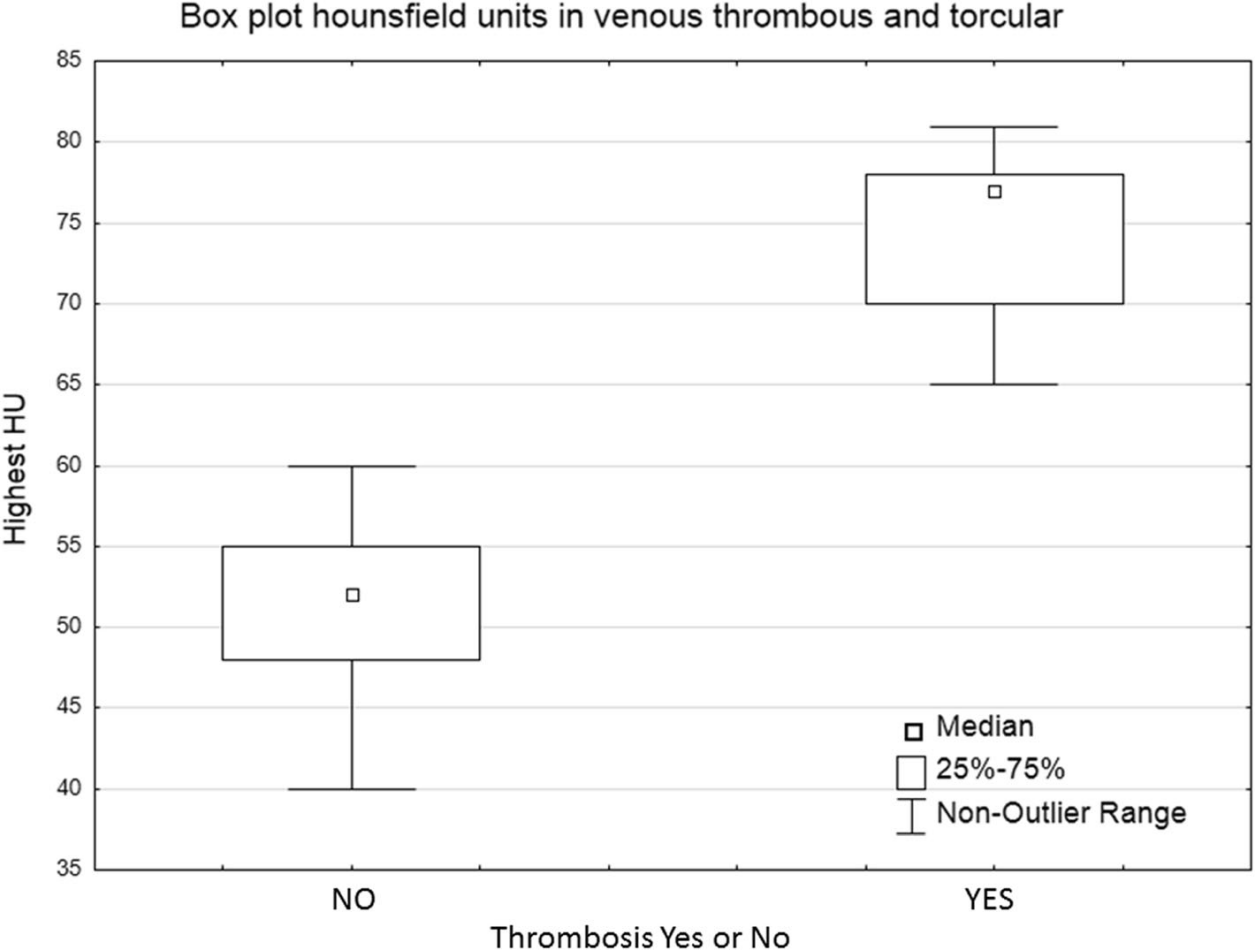
Box plot of hounsfield units measured in the thrombus (maximum HU) and in unaffected torcular herophili

### NeCT and HU in unaffected and thrombosed sinuses, validation cohort

**Fig. 5:**
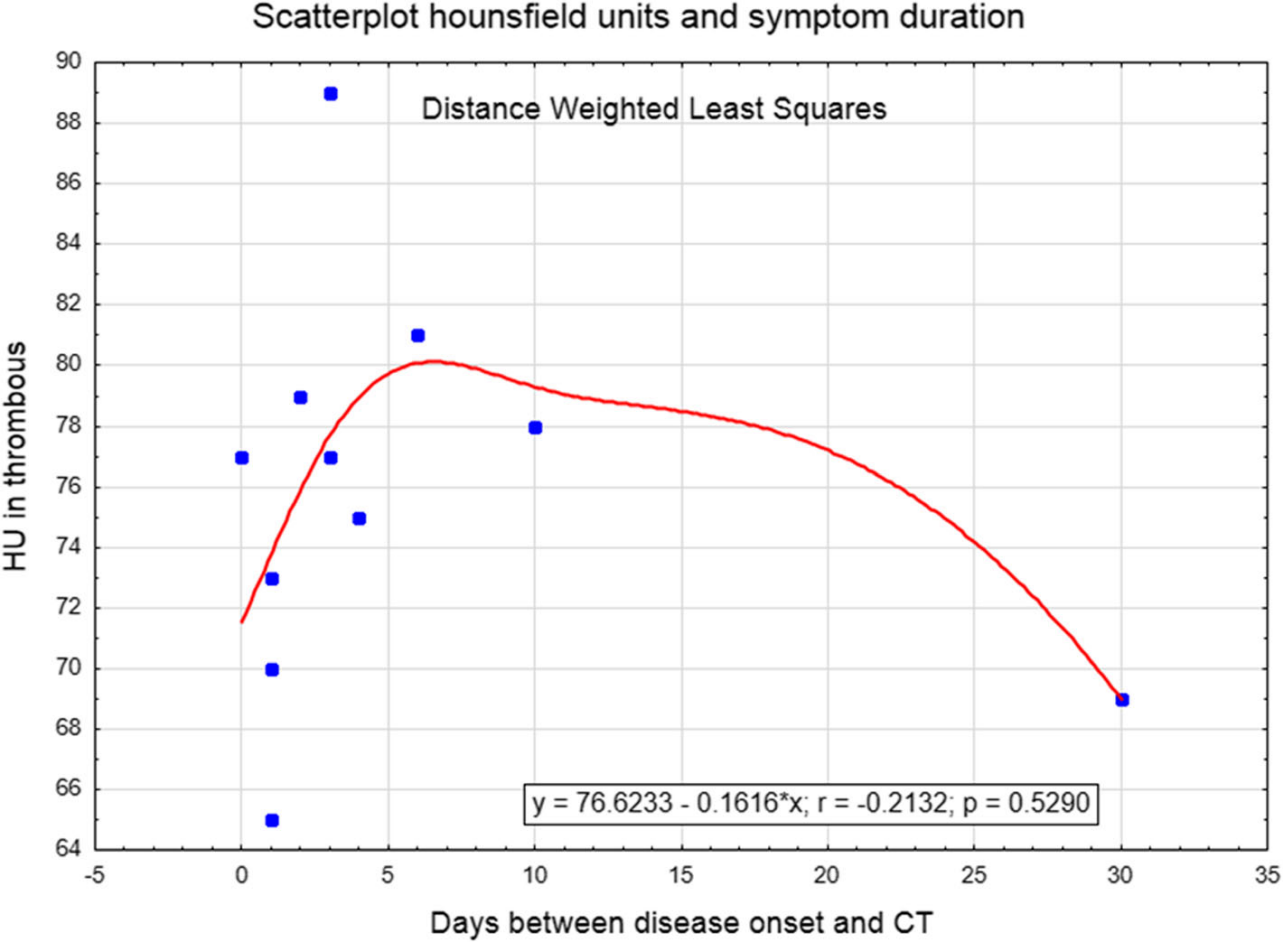
Scatterplot with HU measured in the thrombus (y-axis) and days between symptom onset and neCT (x-axis). A non-linear relationship between symptom duration and thrombus density is noted. HU increases over the first days

In the validation cohort, unblinded reevaluation confirmed the presence of a hyperdense sinus in all patients but one. Including the validation cohort, 11 patients had a venous thrombosis with a mean HU of 75.0 (SD 4.9) measured in the thrombus, compared to 51.7 (4.4) HU measured in the torcular herophili of unaffected individuals (*p* < 0.01) (Fig. 4). Figure 5 describes the non-linear relationship between HU and symptom onset. Receiver operating characteristic analysis using the highest measured HU in the thrombosed region (*n* = 11) against torcular herophili HU in unaffected patients (*n* = 103) revealed maximum accuracy at cut-off HU 65 (AUC = 1). This discriminated between thrombus and normal sinus with 100% sensitivity and specificity.

### CTV, main cohort

All included patients underwent CTV in addition to neCT of the brain. CTV was performed on the same day in 97 patients (93%), in three patients within 1 week, two patients within 2 months and one patient after 8 months and one patient after 22 months. CTV was only positive for CVST in the one patient with suspected CVST on initial neCT (Fig. 2) with thrombus in the left transverse and sigmoid sinus. At a retrospective follow-up of medical charts (median duration of follow-up 29 months, IQR 21–32), three patients were deceased, two from cancer and one due to drug abuse. There were no additional patients diagnosed with CVST or readmitted due to CVST during this follow-up period. The single patient with CVST on CTV in the main study cohort, made a rapid recovery following start of medical treatment, with a complete remission of headache within 2 days. Follow-up CTV after 7 months showed complete restoration of venous flow in the affected sinuses.

### CTV, validation cohort

CTV images were retrospectively assessed for the presence of CVT and confirmed in all cases. One patient was confirmed to have CVST by MRV. All patients in the validation cohort had non-traumatic CVT confirmed by CTV (*n* = 9) or MRV (*n* = 1) (Table 2).

**Table 2:** Clinical and radiological data for validation cohort

ID	Risk factor	Symptom	Duration	CT HU in suspected thrombous	CT HU in nonaffected sinuses	CTV findings	Additional imaging findings	Days between CT-CTV
1	Oral contraception	Headache, vomiting	4 days	75 (SSS), 75 (TS)	44 (TH), 33/38 (SS)	Thrombosed SSS, ISS, TS	0	0
2	0	Loss of consciousness	3 days	77 (cortical vein)	55 (TH), 55 (SSS), 51/54 (TS), 54/49 (SS)	Thrombosed cortical vein	0	0
3	0	Headache	10 days	75 (TH), 78 (SSS), 70/77 (TS), 73/76 (SS)	All affected	Thrombosed SSS, TS, SS	0	0
4	Oral contraception	Headache, vomiting, diarrhea	2 days	79 (vein of Labbé)	51 (TH), 61 (SSS), 55/54 (TS), 51/47 (SS)	Thrombosed vein of Labbé	Focal parenchymal edema (stasis)	0
5	History of deep venous thrombosis	Headache, unilateral arm weakness	1 day	70 (internal cerebral vein)	45 (TH), 47 (SSS), 55/56 (SS), 55/47 (TS)	Thrombosed internal cerebral vein	Focal parenchymal edema (stasis)	0
6	Acute lymphatic leukemia	Unilateral arm weakness, loss of consciousness	1 day	73 (cortical vein), 70 (SSS)	39 (TH), 50/46 (TS), 43/55 (SS)	Thrombosed cortical vein and SSS	Parenchymal hemorrhage in right motor area	0
7	High hematocrit, mutation Factor V Leiden	Seizures	1 day	65 (SSS), 68 (internal cerebral vein), 66 (cortical vein)	49 (TH), 45 (R TS), 50 (R SS)	Massive thrombosis including SSS, L TS, L SS, internal cerebral veins* (*MRI including Phase contrast)	Intraventricular hemorrhage	1*
8	APC	Headache	6 days	81 (SSS), 77 (R TS), 88 (R SS)	56 (TH), 61 (L TS), 57 (L SS)	Thrombosed SSS, R TS, R SS	Parenchymal swelling/general edema	0
9	0	Aphasia	0 days	76 (L TS), 77 (L SS)	55 (TH), 51 (SSS), 58 (R TS), 61 (R SS)	Thrombosed left TS and SS	Parenchymal hemorrhage	0
10	Cerebral metastases	Headache, confusion	> 30 days	69 (SS), 20 (R TS)	36, (TH), 37 (SSS), 39 (LTS), 48 (LSS)	Thrombosed R TS and SS	Cerebral metastasis	0

*APC* Activated protein C, *CT* Computed tomography, *CTV* Computed tomography venography, *F* Female, *HU* Hounsfield unit, *ISS* Inferior sagittal sinus, *L* Left, *M* Male, *R* Right, *SSS* Superior sagittal sinus, *TH* Torcular herophili, *TS* Transverse sinus, *SS*V Sigmoid sinus

### Other imaging findings and final diagnosis, main cohort

In addition to the single patient with CVST, there were eleven additional cases with pathological findings on CT imaging: subarachnoid and subdural hemorrhages were seen in two patients each, meningioma, cerebral metastases, cerebral infarct, arterial aneurysm, and suspected Chiari malformation in one patient each, as well as two cases with fluid levels in the sphenoid sinus indicating sinusitis. Ninety-two (88%) patients had no pathological imaging findings.

### Other imaging findings and final diagnosis, validation cohort

Besides the presence of CVT, two patients had focal parenchymal edema and one patient had generalized parenchymal edema. Three patients had intracerebral hemorrhage and one had cerebral metastasis. The remaining three patients had no additional imaging findings (Table 2).

### Laboratory findings and lumbar pressure, main cohort

Hemoglobin (Hb) ranged from 89 to 176 g/L, mean 135 g/L (SD 16). Hb did not significantly correlate with HU in the superior sagittal sinus (*r* = 0.17, *P* = 0.09) in patients without CVST (*n* = 103). Thirteen patients had been investigated with a D-dimer test, of which 8 had elevated levels (≥ 0.30 mg/L). One of the patients with elevated D-dimer (0.30 mg/L) had confirmed CVST on CTV. The mean levels in patients with elevated D-dimer were 1.8 (3.5) mg/L, range 0.3–10.5. Thirty-one patients had been subject to a lumbar puncture including lumbar cerebrospinal fluid (CSF) pressure measurement. The lumbar pressure ranged from 7 to 50 cm H_2_O. Twenty-one patients had a lumbar pressure above 20 cm H_2_O. In this material, the patient with confirmed CVST did not undergo a lumbar puncture.

### Clinical diagnosis, main cohort

Diagnosis at discharge was unspecified headache in 44% of patients (46/104), tension headache in 19% (20/104), migraine in 8% (8/104), idiopathic intracranial hypertension in 8% (8/104) and one patient each in the following categories: ischemic stroke, depression, post lumbar-puncture headache, reversible cerebral vasoconstriction syndrome, sepsis, sinusitis, dacrocystitis, upper respiratory tract infection, viral meningitis, unspecified virosis, Chiari type 1, intracranial hypotension, cerebral metastasis, jugular vein thrombosis below the level of C2, and two patients with undefined diagnosis.

## Discussion

This retrospective study of imaging findings in patients with nontraumatic headache and a clinical suspicion of CVST, justifies neCT as a primary screening tool for intracranial pathology. In our study, we found no cases of CT venography-verified CVST among patients with normal attenuation of venous sinuses on neCT and symptom duration less than 7 days. In the validation group, one patient with symptoms longer than 30 days had low attenuation (20 HU) in parts of the thrombosed dural sinuses. Our results also advise against using primary CT venography without neCT, since neCT showed other pathological findings in 11% of cases.

The main strength of this study is that it reflects a true clinical situation, including consecutive patients with nontraumatic headache and a clinical suspicion of CVST at one center over 3 years, investigated under similar conditions. The study shows a high incidence of negative radiological investigations. The use of D-dimer as a negative predictor and an adjunct to radiology in patients with suspected CVST has been recommended in the recently published European Stroke Organisation guidelines for the management of CVST [24]. Meanwhile, this recommendation was given with an important caveat regarding patients with isolated headache, or symptoms persisting for more than 1 week before the workup, as both factors have been associated with false-negative D-dimer results [24]. Furthermore, data on HU values from standardized measurements in patients without CVST are presented. Our study gives a reference HU range based on 103 patients with similar symptoms and clinical suspicion of CVST with confirmed negative CTV.

The finding of hyperattenuation in a thrombosed sinus is in accordance with previously reported findings [11, 12, 19–21]. In previously published material different cutoffs for HU have been proposed ranging from 62 to 70 [12, 19–21]. Data of non affected sinuses in this study can aid radiological decision-making when trying to rule-out or lower the suspicion of CVST. The mean HU value in non affected sinuses was 56.9 (SD 5.1) and the optimal cut-off was 65 HU.

Differences in measured mean HU between studies can be explained by differences in population selection, hematocrit level, type and calibration of CT-scanner, and region of interest delineation method. Our results do not indicate a strong correlation between hemoglobin counts and HU in the superior sagittal sinus. However, we found a negative correlation between HU and patient age. This study suggests that primary use of CTV without prior neCT might lead to missed other diagnoses.

Our study is limited by its single-center, retrospective design and the findings would benefit from confirmation in prospective multi-center studies. Our results cannot be generalized to patients with headache after head trauma, nor to patients with recurrent headache after previous CVST, as such cases were excluded. Due to the retrospective nature of the data, we could not reassess the criteria for when the referring physician should ask to rule out or confirm CVST. Only one radiologist measured the HU, limiting the possibility for interobserver variance to be assessed. However, the intraobserver variance of measured HU within normal regions was lower than the difference in HU between CVST and not CVST regions, likely restricting the impact of this limitation. Inclusion criteria differed between the main cohort and the validation cohort allowing to include more patients with CVT in the validation group. The main reasons for a higher proportion of patients with thrombosis in the validation group were: no requirement for the referring physician to suspect CVST in the written referral, no requirement for the patient to present with headache, and the inclusion of deep and cortical CVT, as well as transferred images from other centra.

## Conclusions

Despite clinical suspicion, imaging findings of CVST in nontraumatic headache are uncommon. Evaluating neCT for high attenuation in dural sinuses, followed by CTV for confirmation in selected cases seems reasonable. CVST should be recognized by all radiologists and requires a high level of awareness when reading neCT for other indications.

## Data Availability

Data presented in this article can be made available upon reasonable request to: anna.falk-delgado@neuroradkarolinska.se

## Abbreviations

CT: Computed tomography
CTV: Computed tomography venography
CVST: Cerebral venous sinus thrombosis
CVT: Cerebral venous thrombosis
HU: Hounsfield unit
MRI: Magnetic resonance imaging
neCT: nonenhanced CT
PACS: Picture archiving and communication system
RIS: Radiology Information System
VTE: Venous thromboembolism
